# A 5-year look-back at the notification and management of vaccine supply shortages in Germany

**DOI:** 10.2807/1560-7917.ES.2022.27.17.2100167

**Published:** 2022-04-28

**Authors:** Maria Auxiliadora Miranda-García, Marcus Hoffelner, Hagen Stoll, Dörte Ruhaltinger, Klaus Cichutek, Anette Siedler, Isabelle Bekeredjian-Ding

**Affiliations:** 1 Paul Ehrlich Institut, Langen, Germany; 2 Robert-Koch-Institut, Department for Infectious Disease Epidemiology, Berlin, Germany

**Keywords:** Vaccines, Supply shortage, DTP, Pertussis, Polio, Hepatitis B, Hepatitis A, Pneumococcal polysaccharide vaccines

## Abstract

**Background:**

Unavailability of vaccines endangers the overall goal to protect individuals and whole populations against infections.

**Methods:**

The German notification system includes the publication of vaccine supply shortages reported by marketing authorisation holders (MAH), information on the availability of alternative vaccine products, guidance for physicians providing vaccinations and an unavailability reporting tool to monitor regional distribution issues.

**Aim:**

This study provides a retrospective analysis of supply issues and measures in the context of European and global vaccine supply constraints.

**Results:**

between October 2015 and December 2020, the 250 notifications concerned all types of vaccines (54 products). Most shortages were caused by increased demand associated with immigration in Germany in 2015 and 2016, new or extended vaccine recommendations, increased awareness, or changes in global immunisation programmes. Shortages of a duration up to 30 days were mitigated using existing storage capacities. Longer shortages, triggered by high demand on a national level, were mitigated using alternative products and re-allocation; in a few cases, vaccines were imported. However, for long lasting supply shortages associated with increased global demand, often occurring in combination with manufacturing issues, few compensatory mechanisms were available. Nevertheless, only few critical incidents were identified: (i) shortage of hexavalent vaccines endangering neonatal immunisation programmes in 2015;(ii) distribution issues with influenza vaccines in 2018; and (iii) unmet demand for pneumococcal and influenza vaccines during the coronavirus disease (COVID)-19 pandemic.

**Conclusion:**

Vaccine product shortages in Germany resemble those present in neighbouring EU states and often reflect increased global demand not matched by manufacturing capacities.

## Introduction

The success of immunisation programmes depends on the availability of effective and safe vaccines. Vaccine shortages can negatively affect the success of immunisation programmes and endanger populations with the lowest coverage and the highest risk for infection. Vulnerable patient groups such as pre-term infants, pregnant women, and people with chronic medical conditions (e.g., immunocompromised and elderly individuals) are often under-vaccinated [1]. Thus, access to medicines or vaccines should follow the principle of prioritisation of high-risk groups and, when availability is limited, should consider an ethical framework to ensure equity and fairness along with public health interest [2,3]. COVID-19 vaccination programmes illustrate the difficulties encountered when attempting to abide by these principles [4-7].

In the European Union (EU), market approval for vaccines is granted through the European Medicines Agency (EMA) and the European Commission; however, each Member State is responsible for the official recommendation of a vaccine in its country-specific immunisation schedule and the way it is made available (e.g., via tenders or in an open market). This condition creates some diversity across the EU in regard to demand and stockpiling [8]. In contrast to other EU countries, in Germany, all EU-licensed vaccine products can be made available through the marketing authorisation holders (MAH) and there are neither tenders nor governmental vaccine stocks. With little governmental intervention except when there are shortages, MAH and pharmaceutical wholesale traders are responsible for supply and distribution of vaccines. To date, the total amount of vaccine doses delivered to or stored at pharmacies or with physicians is not centrally monitored. Shortages occur due to disruption of delivery during manufacturing, packaging or any stage of distribution. As unavailability of vaccines could lead to a delay or even omission of vaccinations, national immunisation programmes could be compromised, placing vulnerable populations at increased risk [1]. However, the actual impact of severe shortages is often difficult to assess. Concurrent with an increased global demand, shortages of vaccines and medicinal products have become more frequent, raising concerns of systematic market failures in a product category considered to bring only low return of investment despite complex manufacturing processes [9-14].

Vaccine supply shortages remain critical issues globally, including in most European countries [14,15]. The European Commission and EMA have addressed the threat and issued recommendations for prevention and management of shortages of important medicinal products, which was further supported by the European Council recommendation of 7 December 2018 on strengthened cooperation against vaccine-preventable diseases (2018/C 466/01) [15-18]. There are some important tools for managing and ideally preventing vaccine shortages. In the immediate situation, alternative products and solutions should be made available to those potentially affected by shortages. As in many parts of the world, many European countries have initiated communication practices that inform the public and professionals on vaccine shortages on the national level (Supplement S1, Notification systems in the EU) and [19]. However, data collection and the definition of shortages have not been harmonised. In 2019, the single point of contact (SPOC) was adopted by Human Medicines Agencies (HMA) and the EMA Management Board. The SPOC system intends to improve the collaboration on shortages and availability across the EU regulatory network [20]. The SPOC should contribute to better detection, notification, prevention, management and communication on availability issues [21].

Despite all these efforts, few data have been published on the frequency and impact of vaccine shortages in the EU. Furthermore, the actual impact of shortages and mitigation measures on specific vaccine-preventable diseases remain ill-defined. Here, we explain the current German reporting system for vaccine shortages and the integrated guidance, which was established to mitigate the consequences of the unavailability. This 5-year retrospective analysis of vaccine shortages from October 2015 to December 2020 provides insight into the major causes and dynamics of these shortages as well as the utility of the system.

## Methods

### Notification and management of vaccine shortages in Germany

In 2015, we established a system for public reporting of vaccine supply shortages in Germany. The system is based on MAH providing voluntary information on upcoming supply shortages and the estimated duration of cessation of supply. Notably, the system provides information on the delivery capacity of MAH, not market supply. Supply shortage is defined as an expected interruption of more than 2 weeks of supply at the level of the manufacturer or if an unexpectedly high demand cannot be met. Incoming reports are categorised based on the risk for supply constraints on the market and impairment of the vaccination schedule (Supplement S2, Shortage classification). These reports are published within 24 hours and updated upon notification of the MAH. The results are presented to the MAH, and the system revised yearly.

One unique feature of the system is that it offers solutions to mitigate the consequences of supply shortages. These include listing of alternative vaccine products with the same indication or other packaging sizes. Availability of alternative vaccine products is confirmed by the respective MAH. If alternatives are not available or indications differ between the product in shortage and that being available, the Standing Committee on Vaccination (STIKO) issues guidance for vaccinating physicians. Specifically, STIKO guides physicians on specific risk groups, postpone booster doses, or use vaccine products with similar or extended indications when feasible.

To monitor regional distribution issues, we introduced an electronic form where unavailability of vaccine products (not listed as shortages) is reported together with the pharmacies or wholesalers are contacted (www.pei.de/lieferengpaesse-verbrauchermeldung). Reports are reviewed regularly to detect impending shortages on the regional and national level.

### Comparison with other EU countries

Websites providing information of supply shortages were systematically searched for by EU country (Supplement S1). The shortages and their severity were verified by comparison of supply shortages for vaccine products published in other EU countries. These were searched for and collected via systematic screening of lists and downloads available on the websites of the responsible government organizations and are listed under ‘EU-based comparison of shortage notifications’.

## Results

### Classification and duration of reported shortages

Altogether, 250 supply shortages were reported from October 2015 to December 2020 for 54 vaccine products. These included 124 (49.6%) shortages of certain packaging sizes (category 1, Supplement S2) and 126 (50.4%) product shortages in the other categories (50 category 2 and 76 category 3). The overall frequency of product shortages fluctuated over the years (Figure, panel A): 2015 (only the 4th quarter) (n = 14), 2016 (n = 28), 2017 (n = 29), 2018 (n = 31), 2019 (n = 15) and 2020 (n = 9). The duration of product shortages (category 2 and 3) was on average 134.85 days ± 200 days (SD); 20 (16%) shortages were resolved within 30 days, 60 (48%) shortages lasted between 31 and 100 days, and 46 (36%) shortages lasted more than 100 days (Figure, panel B). All types of vaccines (54 vaccine products) were in shortage once or several times (Figure, panel C). Shortages of certain vaccine types occurred repeatedly such as MMR-V vaccines (Figure, panel D).

**Figure:**
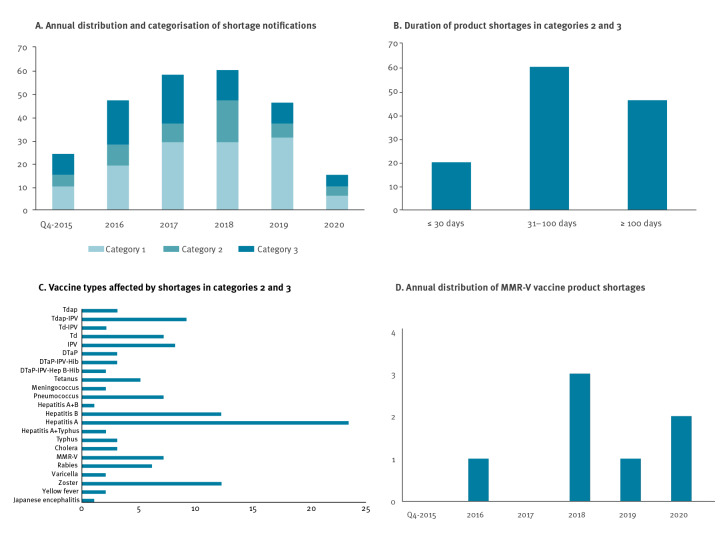
Vaccine supply shortages reported by marketing authorisation holders from October 2015 to December 2020

DTaP: diphtheria, tetanus, acellular pertussis; DTaP-IPV-Hib: diphtheria, tetanus, acellular pertussis, polio, haemophilus influenzae type b; DTaP-IPV-HepB-Hib: diphtheria, tetanus, acellular pertussis, polio, hepatitis B, haemophilus influenzae type b; IPV: polio; MMR-V: measles, mumps, rubella, varicella; Td: tetanus, diphtheria; Tdap: tetanus, diphtheria, acellular pertussis; Td-IPV: tetanus, diphtheria, polio; Tdap-IPV: tetanus, diphtheria, acellular pertussis, polio.

Notably, in 2018, a 38% reduction in shortages for category 3 products was accompanied by an increase in category 2 products; twice as many shortages were notified for category 2 in 2018 than in 2017 (Figure, panel A). In 2019, there was a marked reduction of category 3 shortages; however, the few products reported belonged to those with a long shortage duration. The vaccines subject to shortages more than 100 days between 2016 and 2020 are listed in Table 1. Many of these shortages were labelled with ‘limited availability’ on the PEI shortage website because demand exceeded regularly provided supply. Causes of shortages reported by the MAH are summarised in Table 2. Of note, in Germany, MAH are responsible to forecast demand using market surveys, epidemiological data and subscriptions from previous years. Furthermore, as access to vaccination occurs via physician contacts, patients requiring regular medical care have easier access.

**Table 1:** Vaccines not available for more than 100 days, Germany, 2016–2020 (n = 40)

Vaccine type	2016	2017	2018	2019	2020	Total (n = 40)
Hepatitis A	1	3	2	0	0	6
Hepatitis B	0	2	3	1	0	6
MMR-V	0	0	1	0	1	2
HPV	0	0	0	0	1	1
Td	0	1	2	0	0	3
Tetanus	0	2	2	0	0	4
Typhus	0	1	0	0	0	1
DTaP	1	0	0	0	0	1
DTaP-IPV-Hib	1	0	0	0	0	1
DTaP-IPV-HepB-Hib	0	1	0	0	0	1
Tdap-IPV	2	1	0	0	0	3
IPV	1	2	0	0	0	3
Rabies	0	2	1	0	0	3
Yellow fever	1	0	0	0	0	1
Herpes zoster	0	0	0	1	0	1
Meningococcus	1	0	1	0	0	2
Pneumococcus	1	0	0	0	0	1

DTaP: diphtheria, tetanus, acellular pertussis; DTaP-IPV-Hib: diphtheria, tetanus, acellular pertussis, polio, haemophilus influenzae type b; DTaP-IPV-HepB-Hib: diphtheria, tetanus, acellular pertussis, polio, hepatitis B, haemophilus influenzae type b; HPV: human papillomavirus; IPV: polio; MMR-V: measles, mumps, rubella, varicella; Td: tetanus, diphtheria; Tdap: tetanus, diphtheria, acellular pertussis; Tdap-IPV: tetanus, diphtheria, acellular pertussis, polio.

**Table 2:** Causes for shortages in categories 2 and 3^a^ provided by marketing authorisation holders, Germany, 2017–2020 (n = 81)

Shortages causes	2017	2018	2019	2020	Total	%
Manufacturing issues	3	16	3	1	**23**	28.4
Increased demand	15	13	10	5	**43**	53.0
Both items reported (Manufacturing problems + increase in demand)	5	1	1	0	**7**	8.7
Other issues	6	1	1	0	**8**	9.9
**Total**	29	31	15	6	**81**	100

^a^ Category 2: product shortages with availability of alternative products; category 3: product shortages without availability of alternative products (Supplement S2).

### Shortages trigger supply constraints of other packaging sizes and products

Of the 54 vaccines in shortage, 23 products were only reported once and remained in the category they were first assigned. Five category 1 products became category 2 products and six category 2 products became category 3 products. Thus, cessation of supply of a certain packaging size can precede a product shortage, and a shortage can trigger shortage of alternative products. Notably, resolution of shortages also permitted re-evaluation and downscaling of the shortage category from category 3 to category 2 for seven products. Other changes in category, such as from category 3 to category 1, were less frequently observed.

### Availability reporting

From Q4/2018 to Q4/2020, 8,447 reports were registered. The main users of this reporting tool were pharmacists (56%, n = 4,771), physicians (17%, n = 1,423), and private individuals (27%, n = 2,253). Of the private individuals, 29% (n = 1,257) visited one pharmacy, 16% (n = 722) visited two pharmacies, and 55% (n = 2,442) visited three or more pharmacies. The majority of reports were related to influenza vaccines in the 2018/19 (n = 2,650) and the 2020/21 (n = 5,772) influenza seasons; by contrast, only 11 reports were submitted in the 2019/20 influenza season. In addition, human papilloma virus and herpes zoster vaccine shortages were reported in 2019/20, and a pneumococcal polysaccharide vaccine shortage was reported in 2020 due to the higher demand during the coronavirus disease (COVID-19) pandemic. Notably, these notifications reflected unavailability of certain packaging sizes or known supply constraints, which were classified as ‘limited availability’ because of demand exceeding production capacity.

### Critical vaccine shortages and mitigations

Cessation of delivery at the MAH does not necessarily reflect the market situation. Thus, every shortage requires specific evaluation and measures. Only on three occasions did the German Ministry of Health declare a critical shortage (German medicines legislation, AMG §79 [5]). This legal measure facilitates the import of medicines to prevent or mitigate shortages and was used in three circumstances.

In 2016, there was a temporal shortage of hexavalent vaccines for primary immunisation of neonates (DTP/Polio/Hib/HepB) following a quality issue that delayed production. It was effectively bridged by increasing the market share of an alternative product, which included import of vaccine doses enabled by this legal measure.

During the 2018/19 influenza season, regional distribution problems of influenza vaccines were mitigated by enabling exchange of vaccine doses among physicians and the import of influenza vaccine doses. Consequently, unavailability reports dropped from 2,383 in November 2018 to 44 in January 2019. Notably, in 2020, reports confirmed a high demand for influenza vaccines, but no critical shortage situation was declared because supply issues were affecting many other European countries and import and reallocation were deemed inappropriate measures as they would endanger supply in other regions.

The COVID-19 pandemic was an important stress test for healthcare systems and medicine supply chains. In Germany, crisis-mediated risk awareness drove the demand for vaccines for other respiratory pathogens (e.g., influenza virus and pneumococcus). This demand was in line with the experts’ opinion that the elderly population needed protection from respiratory pathogens [6], but this demand overstrained the market offer. By contrast, worldwide vaccination programmes suffered from lockdown measures and prioritisation of resources [7,22]. As expected, limited production capacity for COVID-19 vaccines led to shortages, prioritisation of risk groups for primary and booster vaccinations, especially elderly people [5,23], and inequity in global distribution [4,24].

Facing an unexpectedly high demand during the COVID-19 pandemic, the Ministry of Health declared a critical shortage for pneumococcal vaccines, which allowed for the import of vaccines with a label in another language than German. However, the measure was less effective than in other occasions because there was no suitable alternative product for the unconjugated pneumococcal polysaccharide vaccine and only limited amounts of vaccine were available for re-allocation to Germany as other countries, including EU countries, were experiencing similar supply issues. Unavailability of unconjugated pneumococcal polysaccharide vaccine still resulted in additional supply shortages of conjugated pneumococcal polysaccharide vaccines despite divergent recommended indications.

### EU-based comparison of shortage notifications

A comparison of vaccine shortage reporting systems in France and the German-speaking countries revealed differences: the French system distinguishes cessation of supply, supply shortage, tension in supply, and resolved shortage; the Austrian system’s website provides supply shortages and limited availability; and the Swiss system’s list is limited to supply shortages. However, all three countries had specific shortages that resembled the shortages experienced in Germany, with exception of BCG and Act-HiB vaccines, which are not marketed in Germany, Austria, or Switzerland (Table 3).

**Table 3:** Comparative analysis of vaccine supply shortages in Germany, France, Switzerland, and Austria, 2016–2020

Vaccine products in shortage				
Year	Germany^a^	France^b^	Switzerland^c^	Austria^d^
**2020**	Pneumococcus MMR-V Herpes zoster	BCG, Cholera, DTaP-IPV-Hib, Hepatitis A, MMR^e^, Pneumococcal polysaccharide vaccine^e^	Pneumococcal polysaccharide vaccine^e^, Polio	Hepatitis B, MMR-V^e^, Pneumococcal polysaccharide vaccine^e^
**2019**	Hepatitis A Hepatitis B Herpes zoster MMR-V Cholera Japanese encephalitis	Act-HiB, BCG, C-type meningitis, Hepatitis B, HPV, MMR^e^, Rabies, TBEV, Yellow fever	Cholera, Polio, Rabies	Hepatitis B,
**2018**	Hepatitis A Hepatitis B Td MMR-V Meningococcus Rabies	MMR^e^, pneumococcal polysaccharide vaccine, Tetanus	Hepatitis B, Polio	C-type meningitis, Hepatitis B, MMR-V,
**2017**	Tdap-IPV Hepatitis A Hepatitis A + Hepatitis B Hepatitis A + Typhus Hepatitis B Typhus DTaP-IPV-HepB-Hib DTaP-IPV-Hib DTaP IPV Td Tetanus Pneumococcus Rabies Tetanus Varicella Herpes Zoster	DTaP, Hepatitis A and B^e^, Polio^e^	Polio	Hepatitis B^e^
**2016**	DTaP DTaP-IPV-HepB-Hib DTaP-IPV-Hib Td-IPV Tdap-IPV IPV Cholera Meningococcus Pneumococcus Hepatitis A MMR-V Yellow fever Rabies Varicella	BCG, Hepatitis A	Polio	No information available

Act-Hib: haemophilus b conjugate; BCG: bacillus Calmette–Guérin; DT: diphtheria, tetanus; DTaP: diphtheria, tetanus, acellular pertussis; DTaP-IPV-Hib: diphtheria, tetanus, acellular pertussis, polio, haemophilus influenzae type b; DTaP-IPV-HepB-Hib: diphtheria, tetanus, acellular pertussis, polio, hepatitis B, haemophilus influenzae type b; HPV: human papillomavirus; IPV: polio; MMR-V: measles, mumps, rubella, varicella; TBEV: tick-borne encephalitis; Td: Tetanus, diphtheria; Tda: tetanus, diphtheria, acellular pertussis; Td-IPV: tetanus, diphtheria, polio; Tdap-IPV: tetanus, diphtheria, acellular pertussis, polio.

^a^ This study.

^b^ https://ansm.sante.fr/S-informer/Informations-de-securite-Ruptures-de-stock-des-medicaments.

^c^ https://www.bwl.admin.ch/bwl/de/home/themen/heilmittel/meldestelle/aktuelle_versorgungsstoerungen.html.

^d^ https://medicineshortage.basg.gv.at/vertriebseinschraenkungen/faces/adf.task-flow;jsessionid=yCO9IbZMWjeMkBtztQsC8w2k0QI-jB9VyyZzZhQ-FZvQ8L51ZKUM!940367347?_id=main-btf&_document=WEB-INF/main-btf.xml&_afrLoop=1977432160706962&_afrWindowMode=0&_afrWindowId=null.

^e^ Shared supply shortages.

## Discussion

The dual reporting system analysed in this study offers transparency in regard to current, upcoming, and past shortages and combines this with vaccine-specific shortage management based on information on availability of alternative products and specific guidance in line with the World Health Organization’s (WHO) recommendations [14]. Guidance and immediate solutions are keys to mitigating the risks associated with shortages, but not all national reporting tools provide these features (Supplement S1). Of note, in Germany, these need to be combined with the unavailability-reporting tool to help identify regional supply and distribution issues. However, active, situation-adapted advertisement of the tool is required for retrieving reliable feedback as experienced in the 2018/19 and 2020/21 influenza seasons. The number of shortages (not the duration) dropped after implementation of the reporting system, but there is no proof that this is associated with its application. The actual impact of shortages on specific vaccine-preventable diseases remains ill-defined. Analysis of the vaccination surveillance data and infection rates is the next step to defining their criticality in terms of endangering public health.

In the EU, there are usually two or three vaccine products with similar indications, but full interchangeability is often not viable. Furthermore, many countries have tenders, which secure supply but restrict availability of alternative products. One advantage of the German market is that shortages can be compensated through competing vaccine products. This may be the reason why Germany, in contrast to other EU countries, was - so far - not forced to change the immunization programmes in response to a vaccine shortage [19]. However, recurring shortages of the full range of products of a certain vaccine type indicate that there is only limited inventory capacity to offset a competitor’s failure to supply the market. In support of this claim, it was recently proposed that neither presence nor absence of tenders prevents supply shortages [10]. Nevertheless, long-term surveillance studies are needed to understand whether a tender system offers an advantage.

A peculiarity of the German shortage notification system is that packaging size is relevant because physicians are reimbursed for holding stocks of vaccines for patients with state insurance, and patients with private insurance are reimbursed for purchasing single doses from a pharmacy. Thus, unavailability of a certain package size triggers undersupply in certain populations. Physicians use the list of published shortages to justify purchase of alternative packaging sizes and related costs.

In Germany, vaccine shortages of up to 30 days are often compensated by wholesaler, pharmacy, and physician stocks, other packaging sizes, or alternative products. However, in 2015/16, 35 of the vaccine product shortages exceeded 30 days, including all DTaP vaccine shortages. These shortages made it necessary to find alternative vaccine products, delay booster vaccinations, and prioritise risk groups. The need for alternative products for primary immunisation beyond the age of 5 years resulted in off-label use because of non-congruent age indications. Subsequent regulatory approval of aligned age indications for several DTaP vaccines facilitated interchangeable use [25]. Delaying booster vaccinations risked that vaccinees would not be able to catch up with their vaccination schedules. Behavioural studies involving physicians facing vaccine supply issues could help identify the measures and communication concepts most effective at preventing delays in vaccine schedules [26,27]. Luckily, paediatric and adolescent vaccination rates in Germany remained stable [28].

Promoting vaccination requires access to vaccines and recognising the difficulties in securing supply and dealing with unprecedented unavailability of vaccines. As reported to GAVI, 65 countries reported at least one national-level stock-out in 2015, and 73 countries reported 131 stock-out events in 2016. Whereas stock-outs were mostly attributed to funding delays [29], in China, limited production, limited governmental batch release testing capacities, and delays in procurement were also considered important obstacles [30]. To avoid constraints and delays, the EU system builds on release of vaccines by Official Member State Control Laboratories (OMCL) [31] and parallel testing by OMCL and manufacturers. Release of vaccines is mutually recognised by other Member States, which reduces regulatory hurdles, and increases batch release capacities (EU Directive for Human Medicines, 2001/83/EC).

Stock-outs for DTP, polio, and BCG-containing vaccines were predominantly reported to GAVI in 2015/16 [32,33], in China in 2017/18 [30] and across the EU [19]. In Germany, the most frequently reported products in shortage were pertussis and polio-containing polyvalent vaccines (n = 30). Shortages of DTaP vaccines in 2015/16 reflected the migration crisis, increased global demand (i.e., switch from whole cell DTwP to acellular DTaP), and delays associated with upscaling of production capacities [13]. Prolonged supply constraints for polio-containing vaccines in 2016/17 (Table 1) were evoked by the switch from oral (OPV) to inactivated polio vaccines (IPV) in many global regions [34]. The global IPV demand more than doubled from 2013 to 2016, a stress that required upscaling of global production capacities and resulted in supply constraints, delaying the switch in many regions beyond 2017 [34-36]. In Germany, physicians were urged not to booster travellers and travellers were urged not to seek boosters.

In agreement with previous reports [2,14,20,37], the most frequent cause for shortages was increased demand on either the national or global level, followed by issues related to manufacturing. Sometimes these were interconnected (Table 2). Manufacturing issues have a strong impact on the availability of vaccines because the same bulk materials are used in the formulation of many vaccine combinations; therefore, the unavailability of a single component results in shortages of all final products. Additionally, long-lasting production cycles do not allow short-term compensatory activities, and changes such as upscaling of manufacturing can pose a risk for continuity of supply.

From 2001 to 2015, the United States (US) Federal Drug Administration (FDA) reported shortages mainly for viral vaccines (58%); in addition to hepatitis A and B, these included rabies and varicella virus-containing vaccines [38]. In Germany, we experienced prolonged periods of shortages of hepatitis A and B vaccines from 2017 to 2019 (Figure, panel C). Existing supply constraints driven by increased global demand were aggravated by large outbreaks of hepatitis A in the US and the United Kingdom (UK) [39,40]. Reallocation of vaccines to affected regions [39] and coincident manufacturing issues with HBsAg-containing vaccines [40-42] triggered supply shortages [25]. By contrast, rabies vaccine shortages (2016 [1], 2017 [4], 2018 [1]) were mitigated with alternative products and emergency stocks for post-exposure prophylaxis. However, a 4-month cessation of delivery of yellow fever vaccine in 2016 could not be avoided [43].

Altogether, our data indicate that the current German system for shortage notifications provides transparency and supports short-term mitigation measures to resolve supply issues. Nevertheless, it cannot revert the root causes nor protect from global increases in demand that need to be met by increased manufacturing capacity.

In 2018, more than 40,000 measles infections and 37 associated deaths [44] increased the demand for MMR-V vaccines in the EU. This coincided with manufacturing issues announced in late 2017, which were responsible for long-term unavailability of the quadrivalent vaccine from one manufacturer. The simultaneity of these events resulted in supply shortages of MMR-V vaccines in several EU countries (Table 3 and Figure, panel D). Moreover, full coverage required administration of trivalent MMR plus monovalent varicella vaccines, which endangered acceptance [45].

Many shortages occurred when new or expanded recommendations or regulations were issued. In 2018, supply issues for influenza vaccines were reported in both Germany and the UK [46]. They were attributable to changes in the UK immunisation programme and the German reimbursement policies. The revision of the STIKO recommendations for pneumococcal vaccination in 2016 triggered increased public awareness and demand for unconjugated pneumococcal polysaccharide vaccines in 2016/17. After the STIKO recommendation of HPV vaccination for boys in 2018 and the introduction of (gender-neutral) HPV vaccination in other countries, Germany experienced supply constraints with repeated stock-outs of individual packaging sizes. The WHO responded to global supply constraints by asking countries to withhold vaccination for boys [47]. Similarly, in 2019, there were shortages of herpes zoster vaccines (n = 8) because the manufacturer could not meet the demand triggered by the recommendation of the subunit vaccine for individuals > 60 years of age. These data underscore that new or extended vaccination programmes can be thwarted by insufficient production capacities. Furthermore, regional differences in regulatory requirements and duration of approval of changes in chemistry, manufacturing and controls (CMC) can create important logistical hurdles [12,13,37] and delay or even prevent reallocation of vaccines to regions with clinical need.

The examples provided highlight that supply shortages endanger public health goals such as elimination of polioviruses, measles and hepatitis B [36,48]. The complexity and duration of manufacturing limits short-term mitigation measures to re-allocation from existing vaccine stocks provided that batches are prepared according to current regional regulatory requirements [12,13]. Re-allocation measures can achieve transient improvement of regional supply (e.g., import of hexavalent vaccines for neonates in 2015 or the influenza vaccine in 2018). However, re-allocation measures remain ineffective in EU-wide shortages triggered by unexpectedly high demand (e.g., MMR-V vaccines in 2018 and influenza vaccines in the 2020/21), or in global supply constraints (e.g., polio and hepatitis A/B vaccines). Therefore, prevention of shortages requires (i) international collaboration to improve demand forecasting and distribution practices, (ii) international harmonisation of regulatory requirements to facilitate re-distribution, and (iii) new technological concepts to stabilise production processes and upscaling to keep pace with global demand [12-14,37,49].

## Conclusions

Reporting of vaccine supply shortages should include reliable information on availability of alternative products and specific guidance for vaccinating healthcare professionals. Management of vaccine supply shortages needs to be tailored to account for national peculiarities and regional needs. Global mismatch of demand and production capacities limits the effectiveness of both immunisation programmes and regional measures for mitigation of vaccine supply shortages.

## Supplementary Data

121000 Supplementary Material 1

## Supplementary Data

80000 Supplementary Material 2
