# Supplementary material for: A 5-year look-back at the notification and management of vaccine supply shortages in Germany
Source: Euro Surveill. 2022 Apr 28;27(17):2100167. doi: 10.2807/1560-7917.ES.2022.27.17.2100167 (PMC9052770; doi:10.2807/1560-7917.ES.2022.27.17.2100167)
Supplement: Supplementary Material 1 [file 21-00167_BEKEREDJIAN-DING_Supplement_S1.pdf]

This supplementary material is hosted by *Eurosurveillance* as supporting information alongside the article 'A 5-year look-back at the notification and management of vaccine supply shortages in Germany' on behalf of the authors who remain responsible for the accuracy and appropriateness of the content. The same standards for ethics, copyright, attributions and permissions as for the article apply. Supplements are not edited by *Eurosurveillance* and the journal is not responsible for the maintenance of any links or email addresses provided therein.

**Supplement S1: Examples for public notification systems on vaccine supply shortages in Europe.**

| Country | Agency | Information of shortages and disruption in the supply chain | Recommendation of alternatives | Publication in English |
|---|---|---|---|---|
| **Austria (A)** | Bundesamt für Sicherheit im Gesundheitswesen<br>https://medicineshortage.basg.gv.at/vertriebseinschraenkungen/faces/adf.task-flow;jsessionid=yCO9IbZMWjeMkBtztQsC8w2k0QI-jB9VyyZzZhQ-FZvQ8L51ZKUM!940367347?_id=main-btf&_document=WEB-INF/main-btf.xml&_afrLoop=1977432160706962&_afrWindowMode=0&_afrWindowId=null | ✓ | ✓ | no |
| **Belgium (BE)** | Federal Agency for Medicines and Health Products<br>www.fagg-afmps.be<br>Shortages list<br>https://pharmastatut.be/human | ✓ | ✓ | no |

| | | | | |
|---|---|---|---|---|
| **Croatia (HR)** | Agency for medicinal products and medical devices of Croatia<br><br>www.almp.hr | ✓ | no | ✓ |
| **Denmark (DK)** | Danish Medicines Agency<br><br>https://laegemiddelstyrelsen.dk/en/licensing/supervision-and-inspection/medicine-shortages/ | ✓ | no | ✓ |
| **France (F)** | Agènce nationale de securité du medicament et des produits de santé<br><br>https://ansm.sante.fr/S-informer/Informations-de-securite-Ruptures-de-stock-des-medicaments | ✓ | ✓ | no |
| **Finland (FI)** | Finnish Medicines Agency<br><br>https://www.fimea.fi/web/en/databases_and_registers/shortages | ✓ | no | ✓ |
| **Hungary (HU)** | National Institute of Pharmacy and Nutrition | ✓ | no | ✓ |

| | | | | |
|---|---|---|---|---|
| | www.ogyei.gov.hu | | | |
| **Latvia (LV)** | State Agency of Medicines<br><br>www.zva.gov.lv | ✓ | ✓ | ✓ |
| **Romania (RO)** | National Medicines Agency<br><br>www.anm.ro | ✓ | no | ✓ |
| **Spain (ES)** | Spanish Agency for Medicines and Health Products<br><br>www.aemps.gob.es | ✓ | no | ✓ |
| **Switzerland (CH)** | Bundesamt für wirtschaftliche Landesversorgung and Bundesamt für Gesundheit<br><br>https://www.bwl.admin.ch/bwl/de/home/themen/heilmittel/meldestelle/aktuelle_versorgungsstoerungen.html and<br><br>https://www.bag.admin.ch/bag/de/home/gesund-leben/gesundheitsfoerderung-und-praevention/impfungen- | ✓ | ✓ | no |

| | | | | |
|---|---|---|---|---|
| | prophylaxe/informationen-fachleute-gesundheitspersonal/impfstoffversorgung.html | | | |
