# Supplementary material for: A 5-year look-back at the notification and management of vaccine supply shortages in Germany
Source: Euro Surveill. 2022 Apr 28;27(17):2100167. doi: 10.2807/1560-7917.ES.2022.27.17.2100167 (PMC9052770; doi:10.2807/1560-7917.ES.2022.27.17.2100167)
Supplement: Supplementary Material 2 [file 21-00167_BEKEREDJIAN-DING_Supplement_S2.pdf]

This supplementary material is hosted by *Eurosurveillance* as supporting information alongside the article 'A 5-year look-back at the notification and management of vaccine supply shortages in Germany' on behalf of the authors who remain responsible for the accuracy and appropriateness of the content. The same standards for ethics, copyright, attributions and permissions as for the article apply. Supplements are not edited by *Eurosurveillance* and the journal is not responsible for the maintenance of any links or email addresses provided therein.

Supplement S2: Categorization of supply shortage notifications and management.

	**Category 1**	**Category 2**	**Category 3**
**Definition**	Constrained supply of individual vaccine packaging sizes	Product supply shortage (all packaging sizes not deliverable), but an alternative vaccine is available for the same or similar indication	Product supply shortage, no alternative product available or the alternative product available is insufficient to cover the demand arising from the shortage
**Action**	Public notification of the shortage on PEI website including<br>- expected duration of shortage	Public notification of the shortage on PEI website including<br>- expected duration of shortage and<br>- alternative products available (confirmation by MAH).	Public notification of the shortage on PEI website including<br>- expected duration of shortage and<br>- STIKO advice for medical prioritization and management<br>- Newsletter to stakeholders
**Stakeholder involvement**	Notifying MAH, PEI	Notifying MAH, PEI,<br>MAH with alternative products<br>RKI/STIKO, if indications differ	Notifying MAH, PEI,<br>MAH with alternative products,<br>RKI/STIKO

MAH: Marketing authorization holders; PEI: Paul-Ehrlich-Institut, RKI: Robert-Koch-Institut; STIKO: German NITAG
